# Perceptions and detection of AI use in manuscript preparation for academic journals

**DOI:** 10.1371/journal.pone.0304807

**Published:** 2024-07-12

**Authors:** Nir Chemaya, Daniel Martin

**Affiliations:** 1 Department of Economics, University of California, Santa Barbara, Santa Barbara, California, United States of America; 2 Kellogg School of Management, Northwestern University, Evanston, Illinois, United States of America; University of Aizu, JAPAN

## Abstract

The rapid advances in Generative AI tools have produced both excitement and worry about how AI will impact academic writing. However, little is known about what norms are emerging around AI use in manuscript preparation or how these norms might be enforced. We address both gaps in the literature by conducting a survey of 271 academics about whether it is necessary to report ChatGPT use in manuscript preparation and by running GPT-modified abstracts from 2,716 published papers through a leading AI detection software to see if these detectors can detect different AI uses in manuscript preparation. We find that most academics do not think that using ChatGPT to fix grammar needs to be reported, but detection software did not always draw this distinction, as abstracts for which GPT was used to fix grammar were often flagged as having a high chance of being written by AI. We also find disagreements among academics on whether more substantial use of ChatGPT to rewrite text needs to be reported, and these differences were related to perceptions of ethics, academic role, and English language background. Finally, we found little difference in their perceptions about reporting ChatGPT and research assistant help, but significant differences in reporting perceptions between these sources of assistance and paid proofreading and other AI assistant tools (Grammarly and Word). Our results suggest that there might be challenges in getting authors to report AI use in manuscript preparation because (i) there is not uniform agreement about what uses of AI should be reported and (ii) journals might have trouble enforcing nuanced reporting requirements using AI detection tools.

## 1 Introduction

There is both excitement and concern about the impact that artificial intelligence (AI) could have on our world. In academia, this has inspired an explosion of research around AI. Some researchers are leveraging AI to help in answering research questions; for example, as a tool for performing statistical or textual analyses (e.g., [1–9]) or for the design or implementation of experiments (e.g., [10–12]). Others have focused more on the interaction of humans with AI systems (e.g., [13–21]) and the larger societal ramifications of AI (e.g., [20, 22–30]).

In terms of societal ramifications, one topic of interest is how AI will impact academia itself, especially given the arrival of powerful Generative AI tools such as ChatGPT and Gemini, which are powered by the emergent abilities of Large Language Models (LLMs). Most of this research has centered on student use of AI to complete course or degree requirements (e.g., [31–38]). However, there has been less focus on the impact that AI is having and might have on manuscript preparation for academic journals, even though the impact this could have on science, and the propagation of scientific results, is potentially large.

An exception is [39], who documents several use cases for LLMs for researchers, including academic writing. The potential impact of these tools is validated by a growing industry that helps academics leverage LLMs in their writing. Some of those initiatives are even run by professors from academia, such as online workshops that teach researchers how to use ChatGPT for academic publishing.

However, a growing set of papers (e.g., [38, 40–42]) detail some practical and ethical concerns with the use of these tools in preparing manuscripts for academic journals. For example, AI can generate a text with mistakes, including incorrect math, reasoning, logic, factual information, and citations (even producing references to scientific papers that do not exist). There are many well-documented examples where LLMs “hallucinate” and provide completely fictitious information. On top of this, these tools may also produce text that is biased against particular groups. These issues are exacerbated by the “black box” nature of LLM suggestions, meaning that we lack an understanding of how they work (e.g., [43]). Given these issues, liabilities arise when authors submit papers without fully vetting the text generated by LLMs. In addition, given that they are trained on a corpus of other writing and not the author’s own writing, using the output of tools like ChatGPT without proper attribution could be considered plagiarism.

Yet to the best of our knowledge, there has been no systematic study of whether academics view it as necessary to report AI use in manuscript preparation or if AI detectors can detect AI use in manuscript preparation. We address both gaps in the literature by conducting a survey of academics about AI use in manuscript preparation and by running AI-modified abstracts from published papers through a leading AI detection software.

We have three main findings. First, most of the academics we surveyed thought it was unnecessary to report using ChatGPT to fix grammar, but detection software did not always draw this distinction, as abstracts for which we used GPT to fix grammar were often flagged as having a high chance of being written by AI. Second, we found disagreements among the academics we surveyed on whether using ChatGPT to rewrite text should be reported, and differences were related to perceptions of ethics, academic role, and English language background. Third, we found little difference in perceptions for reporting ChatGPT and research assistant (RA) help, but significant differences in reporting perceptions between these sources of assistance and paid proofreading and other AI assistant tools (Grammarly and Word).

Our findings are a starting point for future research and suggest that several issues need to be carefully considered by the academic community. For instance, which forms of assistance should be reported, whether it be ChatGPT or some other source, such as RA help or help from another AI tool. Additionally, which types of assistance to be reported, be it fixing grammar or something more extensive. One avenue could be to disclose the actual prompts that are used to revise the paper. Along these lines, Grammarly has a new feature that allows users to acknowledge the usage of AI and the actual prompts that the users used.

There is also the question of whether this disclosure should be voluntary or mandatory. Because of well-documented failures of disclosure in the field and lab (e.g., [44, 45]), journals, conferences, and associations may respond by mandating disclosure through reporting requirements, as is already done for conflict-of-interest issues. For example, Elsevier requires that the use of AI be disclosed at the very end of the paper (before references are listed). See the S2 File for a list of major publishers who have voluntary and mandatory disclosure policies for AI use.

To enforce these reporting requirements, journals, conferences, and associations might turn to using detection services, as many teachers have done with student writing in class settings. However, our research raises the question of how journals, conferences, and associations can enforce differences in what should be reported. While the detection tool that we employed was able to detect relatively accurately whether AI was used at all, abstracts that were rewritten by GPT-3.5 were sometimes given a lower chance of being written by AI than the grammar-fixed abstracts.

These tools and our perceptions of them will surely evolve, but the aim of this paper is to determine how they are perceived and detected in this moment in time, as it appears to be an inflection point in AI ability and in its use to revise text.

## 2 Methods

### 2.1 Survey design

Our survey was conducted using Qualtrics, and survey respondents gave informed consent by clicking a button to proceed with the survey after reviewing the consent form (see the S1 File). In addition, our survey was deemed exempt from the Federal Regulations at 45 CFR 46.101(b) by the Human Subjects Committee at the University of California, Santa Barbara (protocol number 11–22-0691).

We focused on two main aspects related to perceptions of AI use in manuscript preparation in our survey. The first is whether authors should acknowledge using ChatGPT to fix grammar or to rewrite text. We elicited these perceptions over two separate questions, as shown in the S1 File. Given that we are the first to elicit these perceptions from academics, these questions have not been previously validated with the population of interest. We required an answer to all questions so that the data would be complete.

We focused on these two uses of AI (fixing grammar and rewriting text) because they are important intermediate cases between not using AI at all and using AI to do all of the writing based on limited inputs (e.g., only a title and/or a set of results). These two uses of ChatGPT also reflect to many of the use cases of LLMs suggested in articles, websites, and online courses.

The second aspect we focused on is whether using ChatGPT to modify an academic manuscript is unethical. Again, we split this into two questions—one for grammar and one for rewrite—to allow researchers to address whether ethics also depends on usage. Because we suspected that there might be differences in these perceptions and an academic’s role and language background, we asked respondents if they were a native speaker of English and their current role (postdoc, student, untenured professor, tenured professor, and/or other).

In addition to these questions, we added follow-up questions on a second page of the survey about whether authors should acknowledge other writing services and tools such as Word, Grammarly, proofreading, and the work of research assistants (see the S1 File). We asked this question only after the ChatGPT questions and on a new page because our main focus was the perception of AI (e.g., ChatGPT) in academic writing, and because we wanted to avoid these follow-up questions influencing the answers given about ChatGPT. In addition, we did not randomize the order between ChatGPT and other services because the convenience sample already knew this was a survey about ChatGPT, so that would have been in their mind already when completing the survey. However, by asking questions about ChatGPT first, we might have reduced differences in reported perceptions with other writing services, as people might feel the need to report consistently for a given use.

To make the questions about other sources comparable the questions about ChatGPT, we used the same question format and split the comparison into two groups: fixing grammar and rewriting text. The tools we included for fixing grammar included Word, Grammarly, and RA help. Importantly, we wrote explicitly in the question that these tools were to be used for fixing grammar. For rewriting text, we used proofreading and RA help. Once more, we explicitly asked whether authors should acknowledge using these tools and services for rewriting text for an academic journal.

### 2.2 Detection design

We also tested how an AI detector would react to using ChatGPT to fix grammar in academic text and to rewrite academic text. We first collected titles and abstracts from 2,716 papers published in the journal *Management Science* from January 2013 to September 2023. We excluded articles with the following words in their titles, as they did not appear to be original articles: “Erratum,” “Comment on,” “Management Science,” “Reviewers and Guest Associate Editors,” and “Reviewers and Guest Editors.” We intentionally included papers that were published before the launch of ChatGPT in November 30, 2022 in order to have source text that was plausibly unimpacted by LLM use.

We revised these abstracts using a variety of prompts, as seen in Table 1 below. Rather than selecting the prompts ourselves, we wanted to use an external source. We decided to use prompts from the online GitHub page *ChatGPT Prompts for Academic Writing*, which advises researchers on how to use ChatGPT. We chose this source because it was the first link returned from a Google search of “ChatGPT Prompts for Academic Writing.”

**Table 1:** Prompts used to revise abstracts with GPT-3.5.

	Prompt
Grammar 1	Correct the grammar. Give a version in one paragraph based on this paragraph: “[PARAGRAPH]”
Rewrite 1	Rewrite this paragraph in an academic language. Give a version in one paragraph based on this paragraph: “[PARAGRAPH]”
Grammar 2	Act as a language expert, proofread my paper on “[TITLE]” while putting a focus on grammar and punctuation. Give a version in one paragraph based on this paragraph: “[PARAGRAPH]”
Rewrite 2	Improve the clarity and coherence of my writing. Give a version in one paragraph based on this paragraph: “[PARAGRAPH]”
Grammar 1b	Correct the grammar: “[PARAGRAPH]”
Rewrite 1b	Rewrite this paragraph in an academic language: “[PARAGRAPH]”

Our baselines prompts were “Grammar 1” and “Rewrite 1,” and for robustness we also considered two functionally related prompts, “Grammar 2” and “Rewrite 2.” We added a restriction at the end of these prompts to “Give a version in one paragraph based on this paragraph” in order to meet the requirement that the abstract be only one paragraph in length. To consider the robustness of our results to this additional language, we also ran versions of our baseline prompts that did not include it: “Grammar 1b” and “Rewrite 1b.”

It has been shown that there are ways to fool certain detectors, such as by adding strange characters to the text, and this is a constantly evolving game of cat-and-mouse as detectors evolve. Although this could be of interest for researchers, we do not study this particular phenomenon. Instead, it is our goal to see whether an academic who uses ChatGPT for widely-proposed purposes—without further edits or detection avoidance strategies—would have their writing be flagged as AI generated.

To revise these abstracts at scale, we leveraged the GPT API with settings that would produce output that closely mirrors the output of a researcher using ChatGPT to revise academic writing. The specific model we used was gpt-3.5-turbo-0613 (GPT-3.5 Turbo released in June 13 2023), and we kept the default settings for temperature, top p, frequency penalty, and presence penalty. We also used the default *system* prompt, “You are a helpful assistant,” which is a background prompt that can be changed in the API but not in ChatGPT itself. We did a fresh call each time we used a prompt to avoid learning and history effects.

Finally, we used a leading paid service (Originality.ai) to see how AI detection algorithms might react to this use of LLMs. We first evaluated the original *Management Science* abstracts, and then the abstracts revised by GPT-3.5 based on all of the prompts. Originality.ai provides an “AI score,” which is a value between 0% and 100% that is interpreted as the likelihood that AI wrote the text being evaluated. A high score means a high likelihood that AI generated the text. Specifically, the company states: “If an article has an AI score of 5%… there is a 95% chance that the article was human-generated (NOT that 5% of the article is AI generated).” [46] studied a number of popular detection tools and found that Originality.ai had the highest accuracy rate (97%).

## 3 Results

### 3.1 Survey responses

Beginning August 22nd 2023 and running until September 20th 2023, we sent our survey to a convenience sample of academics; specifically, we sent it to three listservs: the UCSB Economic Department listserv (connecting graduate students, professors, and postdocs at the University of California, Santa Barbara); the Economics Science Association (ESA) announcement listserv (ESA is the leading organization for experimental economics); and the Decision Theory (DT) Forum listserv (the DT Forum is a listserv of academics working on decision theory that is run by Itzhak Gilboa). We sent the survey with a few days delay between each group, which allows for a rough measure of the respondents from each source. We had a total of 271 respondents complete the survey: 38 from UCSB, 199 from UCSB or the ESA community, and 34 from UCSB, the ESA community, or the DT Forum (this number does not include 20 individuals who did not specify an academic role). The median time to answer the survey was 1.21 minutes.

Of the 271 respondents who completed our survey, 83 reported being a native English speaker, and we categorized 67 respondents as students, 32 as postdocs, 59 as untenured professors, and 113 as tenured professors. If someone reported multiple roles, we took the “highest” report, ranked in this order.

### 3.2 Fixing grammar: Reporting and detection

**Fig 1:**
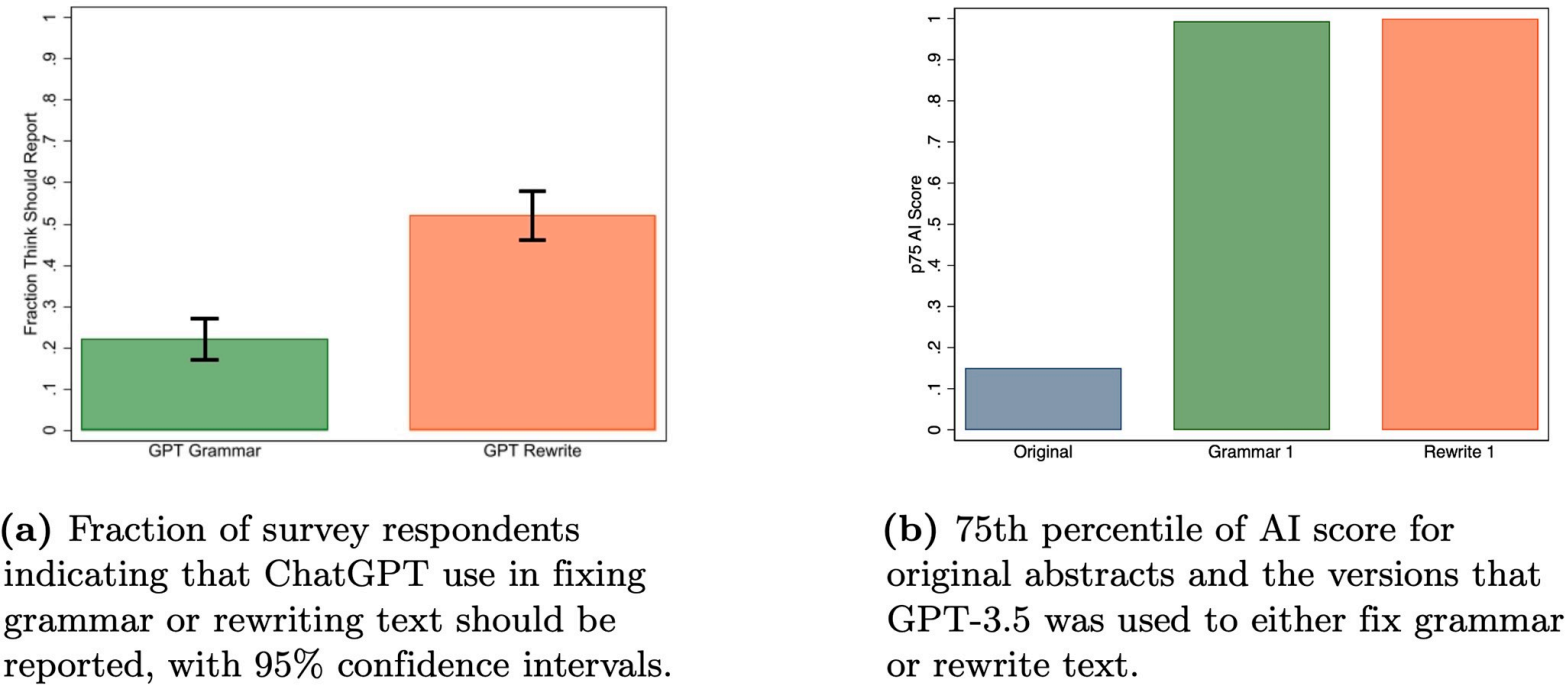
Reporting views (a) vs. detection results (b).

Our initial set of results pertain to using ChatGPT to fix grammar. Looking first at academic perceptions, Fig 1 presents the fraction of survey respondents who indicated that using ChatGPT to fix grammar or rewrite text should be acknowledged. We find substantial differences in reporting views between using ChatGPT for fixing grammar and using it to rewrite text, with 22% of the respondents indicating that grammar correction should be reported relative to 52% for text rewriting (a two-sided test of proportions gives *p* < 0.0001). These perceptions were cleanly nested, as 95% of respondents who thought grammar should be reported also thought rewriting should be reported (only 3 respondents thought that fixing grammar should be reported but not rewriting.).

**Fig 2:**
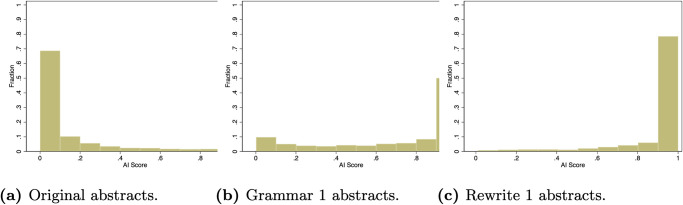
The distribution of AI scores for the original abstracts (a), abstracts revised using the Grammar 1 prompt (b), and abstracts revised using the Rewrite 1 prompt (c).

On aggregate, survey respondents viewed these types of AI use differently, but did the AI detector we study treat them differently? Fig 2 shows that the distribution of AI scores is skewed more to the right for abstracts revised using the Rewrite 1 prompt than for those revised using the Grammar 1 prompt. However, both Grammar 1 and Rewrite 1 produce a large number of high AI scores, and if we look again in Fig 1, the 75th percentile values of the AI scores for the abstracts produced by these two prompts are both near the maximal value. It is worth noting that while the detector gave abstracts revised using both prompts high scores, gave much lower scores to the original abstracts. Thus, it had a high degree of accuracy in separating manuscripts that were not revised by GPT-3.5 from those that were revised in some way.

However, this analysis does not indicate how Grammar 1 and Rewrite 1 compare for a given abstract. It could be that abstracts always had higher AI scores when the Rewrite 1 prompt was used. Looking at the abstract level, 2.2% of abstracts had the same AI score for both types of prompts and 24.2% of abstracts had an higher AI score when the Grammar 1 prompt was used than when the Rewrite 1 prompt was used.

These results combine to produce our first main finding:

**Main Finding 3.1** *Most of the academics we surveyed did not think that it was necessary to report using ChatGPT to fix the grammar in manuscripts, but detection software did not always draw this distinction, as abstracts for which GPT-3.5 was used to fix grammar were often flagged as having a high chance of being written by AI*.

### 3.3 Disagreement about rewriting text

While there was a high level of agreement among the academics we surveyed on whether fixing grammar should be reported, academics were nearly evenly split about whether using ChatGPT to rewrite text should be reported. Next, we investigate the potential reasons why some academics we surveyed thought that using ChatGPT to rewrite text should be reported and why others did not.

**Fig 3:**
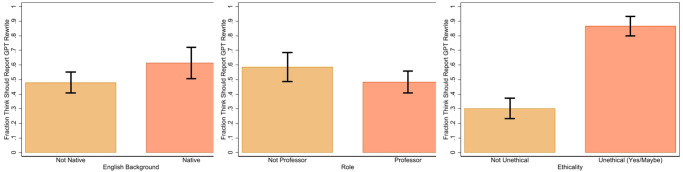
Fraction of survey respondents indicating that ChatGPT use in fixing grammar or rewriting text should be reported, with 95% confidence intervals, by English background, academic role, and perceptions of ethics.

Fig 3 shows how reporting perceptions differ by English language background (native speaker or not), academic role (professor or not), and perceptions of ethics (whether using ChatGPT to rewrite text is unethical or not). To increase statistical power, we collapse role into professor or not and pool together those who answered either “yes” or “maybe” to the survey question on the ethics of using ChatGPT to rewrite text. When we compare native English speakers to non-native ones, we find that on average native speakers are more inclined towards reporting the use of ChatGPT for rewriting text. When we compare professors (both tenured and untenured) to students and postdocs we find that, on average, postdocs and students are more likely to believe that authors should acknowledge the use of ChatGPT when rewriting text. Finally, we find large differences in reporting perceptions based on perceptions of ethics. Survey respondents who believe that it is unethical to use ChatGPT to rewrite text are almost three times more likely to believe that it should be reported. A more detailed analysis of perceptions of ethics is provided in the S2 File.

When we run an OLS regression analysis with only English language background (dummy variable Native = 1) and academic role (dummy variable Professor = 1) as the explanatory variables, as presented in first column of Table 2, we find that the coefficients are significant at the 5% and 10% level for Native and Professor respectively. However, when we add perceptions of ethics to the regression, the effect for Native becomes weaker and less significant.

**Table 2:** Regressions of whether using GPT to rewrite should be reported (dummy variable) onto being a native English speaker or not (dummy variable), being a professor or not (dummy variable), and in the second specification, whether using ChatGPT to rewrite text is unethical or not (dummy variable).

	(1)	(2)
	Report GPT Rewrite	Report GPT Rewrite
Native	0.142**	0.0851
	(0.065)	(0.055)
Professor	-0.111*	-0.102*
	(0.063)	(0.052)
Unethical		0.557***
		(0.052)
Constant	0.547***	0.343***
	(0.053)	(0.048)
*N*	271	271

Note: Standard errors in parentheses.

* *p* < 0.10,

** *p* < 0.05,

*** *p* < 0.01.

**Main Finding 3.2** *We found disagreements among the academics we surveyed about whether using ChatGPT to rewrite text needs to be reported, and differences were related to perceptions of ethics, academic role, and English language background*.

### 3.4 Source of the assistance

Thus far, we have looked exclusively at perceptions of ChatGPT use. However, it is natural to ask academics feel about reporting other sources of assistance for fixing grammar and rewriting text.

**Fig 4:**
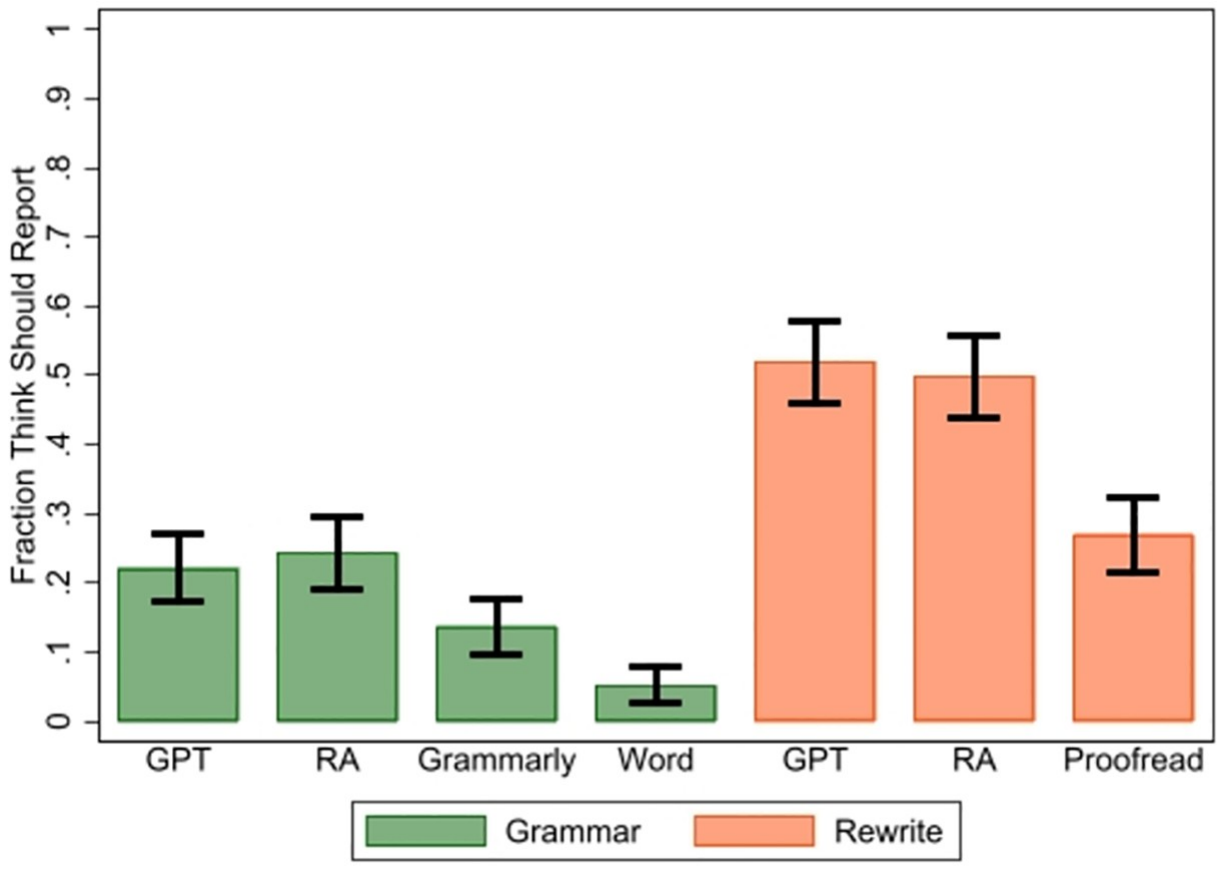
Fraction of survey respondents indicating that ChatGPT, RA, Grammarly, and Word use in fixing grammar or ChatGPT, RA, or Proofreading use in rewriting text should be reported, with 95% confidence intervals.

When we compare our survey results for different sources of assistance, we find very similar reporting perceptions between ChatGPT and the help of a research assistant (RA) for grammar correction or for rewriting text, which is illustrated in Fig 4. More detailed summary statistics are provided in the S2 File. Comparing the difference between RA and ChatGPT for fixing grammar and rewriting text, we find no significant difference (*p* = 0.5418 for fixing grammar and *p* = 0.6062 for rewriting for two-sided tests of proportions).

However, respondents indicated that other tools used for fixing grammar, such as Grammarly and Word, should be acknowledged at even lower rates than ChatGPT, even though these tools might provide users with similar grammar corrections as ChatGPT. Only 14% (5%) of those completing the survey responded that researchers should report using Grammarly (Word) to fix grammar for academic text. A two-sided test of proportions between reporting ChatGPT and Grammarly (Word) for fixing grammar gives *p* = 0.0100 (*p* < 0.0001).

Finally, only 27% of those completing the survey responded that proofreading services should be acknowledged for rewriting academic text, which is much lower than for ChatGPT (52%) and for RA help (49.6%). A two-sided test of proportions comparing ChatGPT to proofreading for rewriting text gives *p* < 0.0001.

This leads to our third main finding:

**Main Finding 3.3** We found little difference in perceptions for reporting ChatGPT and RA help, but significant differences in reporting perceptions between these sources of assistance and paid proofreading and other AI assistant tools (Grammarly and Word).

### 3.5 Detection robustness

Finally, we examine the robustness of our detection results by considering variations on the prompts that we used to fix grammar and rewrite text.

**Fig 5:**
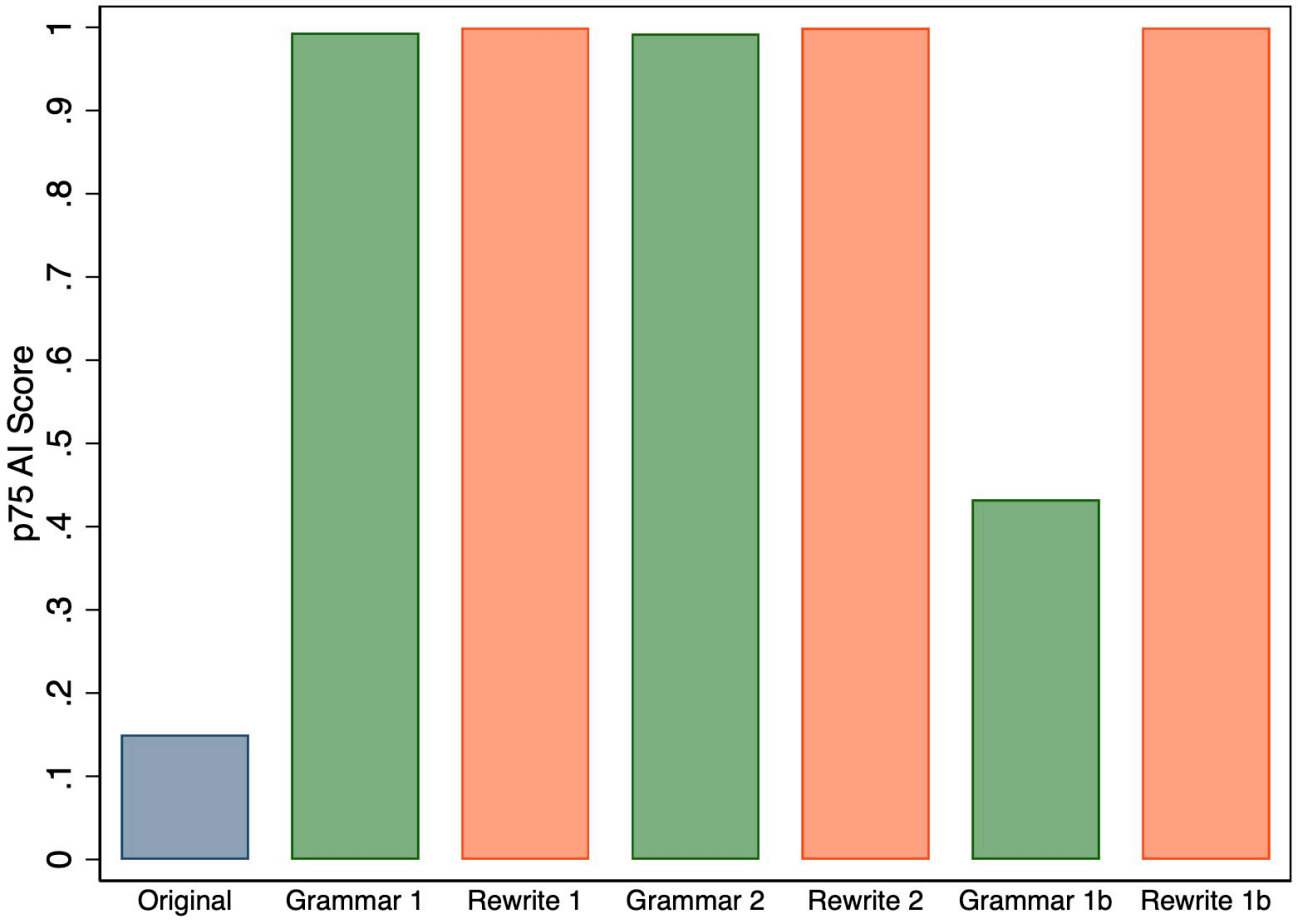
75th percentile of AI score for original abstracts and the versions that were revised by GPT-3.5 for all of the prompts.

**Fig 6:**
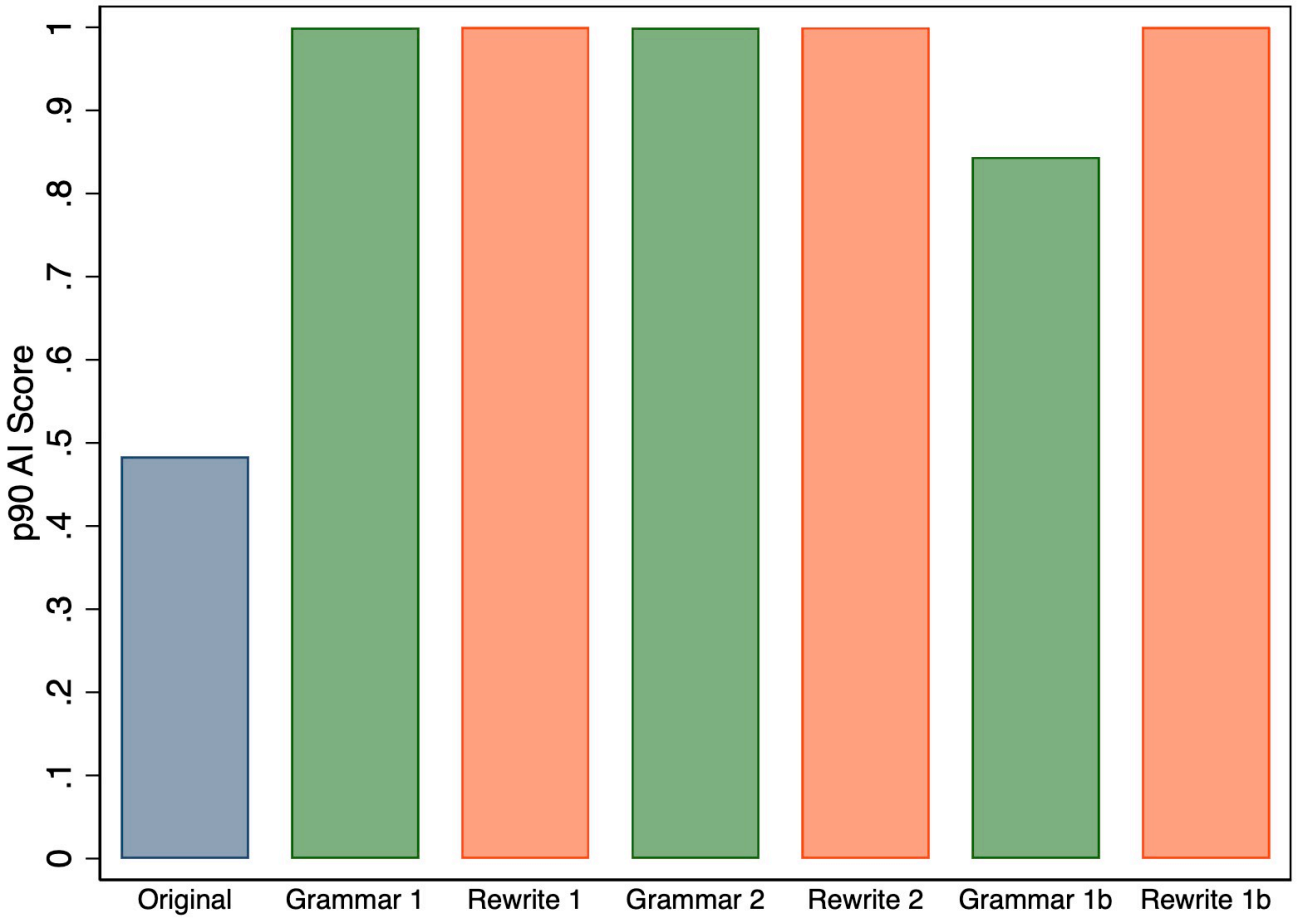
90th percentile of AI score for original abstracts and the versions that were revised by GPT-3.5 for all of the prompts.

First, we compare the 75th percentile of AI scores across all of the prompts provided in Table 1, and Fig 5 shows that we find no perceptible differences for Grammar 2 and Rewrite 2. However, we find that dropping the requirement that the GPT-3.5 output be only one paragraph dramatically reduces the 75th percentile value when revising grammar (Grammar 1b). However, the result is more robust if we consider the 90th percentile instead, as shown in Fig 6.

**Fig 7:**
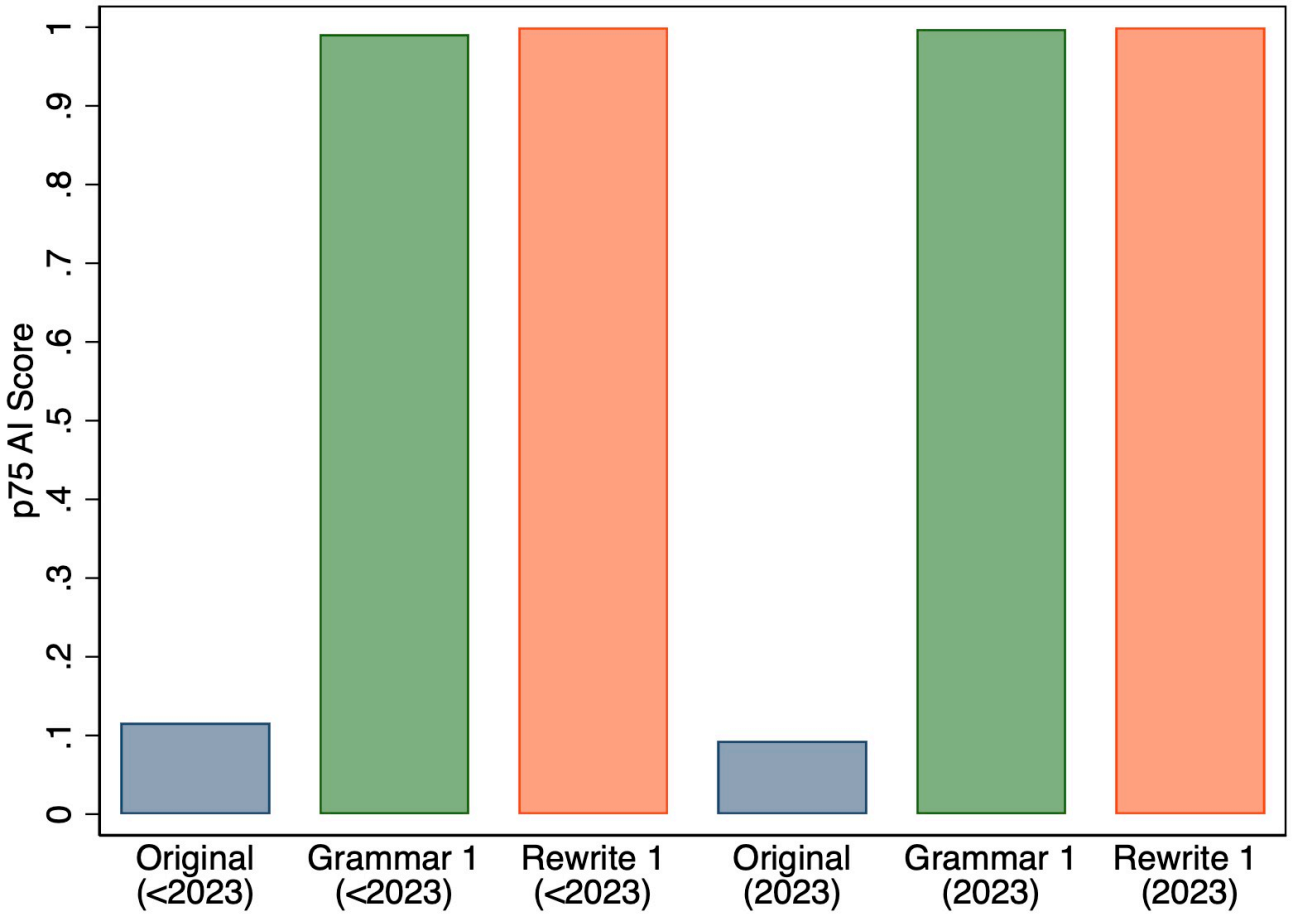
75th percentile of AI score for original abstracts and the versions that GPT-3.5 was used to either fix grammar or rewrite text for abstracts appearing in print before and after 2023.

Second, we checked if the 75th percentile values of the AI scores were different in the years before and after the launch of ChatGPT. Looking at the 75th percentile of AI scores in Fig 7, we find a very slight *decrease* in the 75th percentile AI scores for original abstracts that appeared in 2023. Given this small change for original abstracts, it might not be surprisingly that we do not see much of a difference for Grammar 1 or Rewrite 1.

**Fig 8:**
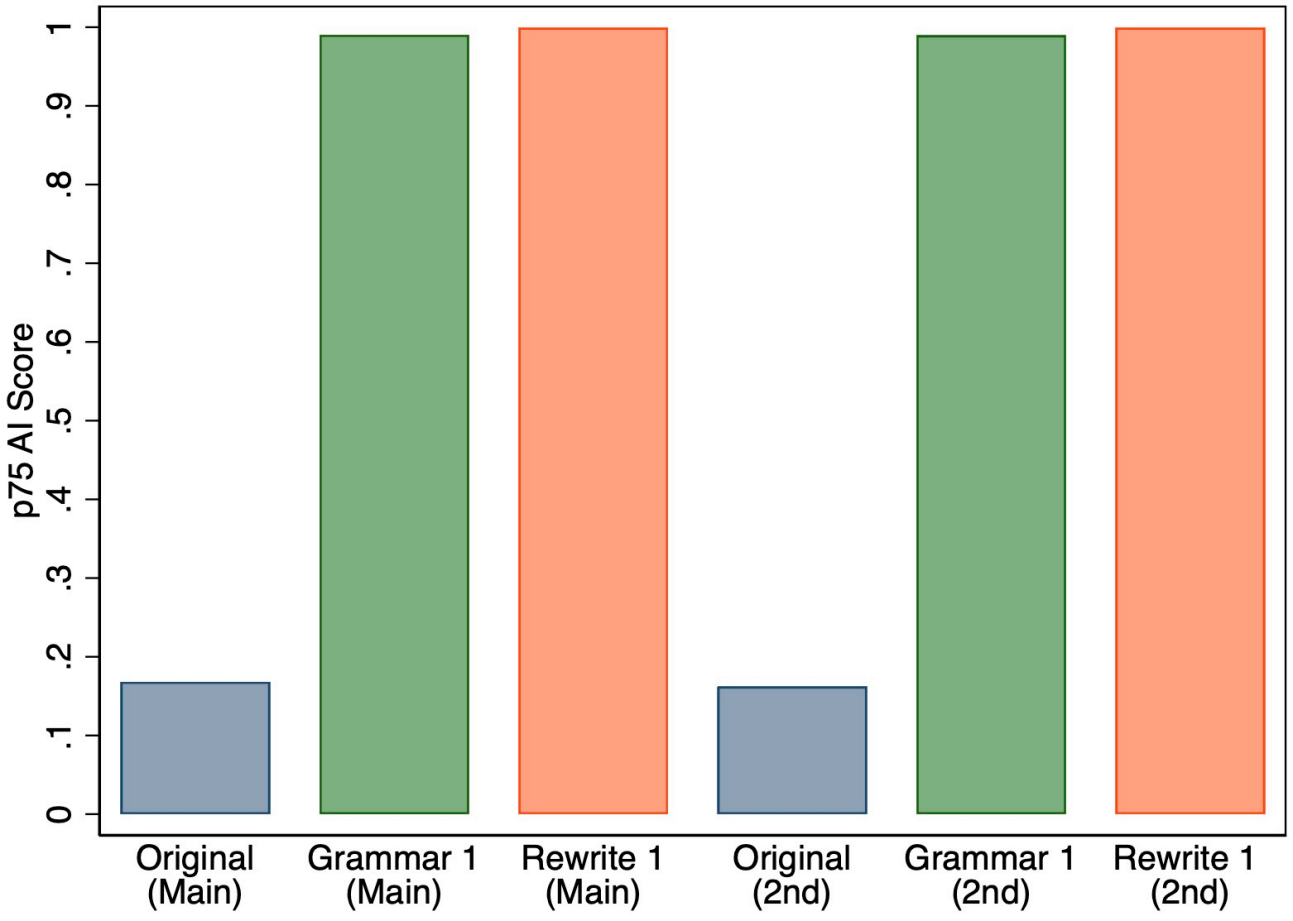
75th percentile of AI score for original abstracts and the versions that GPT-3.5 was used to either fix grammar or rewrite text for abstracts in our main run and in our secondary run.

Third, we ran 1,016 of our abstracts through ChatGPT twice to test for the variability in AI scores due to any stochasticity in ChatGPT. Fig 8 shows that there is very little difference in the 75th percentile of scores.

## 4 Discussion

The first part of our first main finding is that most of the academics we surveyed did not think that it was necessary to report using AI to fix the grammar in manuscripts. This is consistent with the idea that fixing grammar does not substantially change the content of the manuscript, but helps in the communication of scientific findings. It is also in line with many journal policies. For example, Elsevier limits the use of AI by authors “only to improve the language and readability of their paper.”

However, the second part of our first main finding is that the detection software did not always draw this distinction, as abstracts for which GPT-3.5 was used to fix grammar were often flagged as having a high chance of being written by AI. This opens the question of whether ChatGPT corrects grammar in a way that is very “AI-like” or whether using ChatGPT to fix grammar might inadvertently make more substantial changes than desired. How can researchers be sure that they use AI in the desired way? One solution could be to use ChatGPT to point out grammar errors, but to fix them manually, so that ChatGPT does not actually revise the text.

Our second main finding is that we found disagreements among the academics we surveyed about whether using ChatGPT to rewrite text needs to be reported, and differences were related to perceptions of ethics, academic role, and English language background. One possible explanation for why non-professors were *more* likely to think it should be reported is that early career researchers are more conservative because they are unfamiliar with the norms in the profession and prefer to take the safer option and report. On the other hand, they may have a better sense of the power of these tools, and hence might feel that reporting is more necessary.

Another finding is that non-native English speakers are less likely to think that using ChatGPT to rewrite text needs to be reported. When we control for both English language background and perceptions of ethics, the relationship between being a non-native English speaker and perceptions of reporting becomes weaker and less significant. This suggests that some of this effect was due to differences in perceptions of ethics between native and non-native speakers of English.

Our third main finding is that there is little difference in perceptions for reporting ChatGPT and RA help, but significant differences in reporting perceptions between these sources of assistance and paid proofreading and other AI assistant tools (Grammarly and Word). One explanation for the difference with proofreading could be that proofreading might be considered to be closer to fixing grammar than rewriting text (even that we explicitly stated that proofreading would be used for rewriting text). Another potential explanation could stem from an academic norm related to acknowledging this service or the fact that this service is paid. A possible explanation for the difference in perceptions between ChatGPT and other AI assistant tools (Grammarly and Word) is that researchers are unfamiliar with the differences between these tools, especially given that Grammarly uses some AI models ([47]). Alternatively, respondents may be so familiar with those tools that they are less worried about their influence on academic writing compared to ChatGPT, which is relatively new.

### 4.1 Limitations and future directions

In terms of our survey design, the use of a convenience sample may have introduced selection issues that complicate our comparisons by role and English language background. In addition, our convenience sample was largely composed of economists, and because views might differ across fields, it would be valuable to also consider what perceptions look like more generally and to illuminate differences across fields. An alternative approach that allows for an assessment of perceptions across fields is offered by [48], who runs a sentiment analysis on papers written about AI use in manuscript preparation and finds that the sentiment in those papers is generally positive. Also, the use of a convenience sample did not lend itself to randomizing on the form of assistance (ChatGPT, RA assistance, etc.).

In addition, another limitation of our survey is that we do not dig deeply into the nature of ethical perceptions. Since these perceptions were such an important predictor of reporting perceptions, it might be insightful to know why academics feel that using AI tools for manuscript preparation is unethical. For example, is someone harmed by their use—such as other academics, science in general, or the authors whose material is used in training the AI—or is there a deeper moral question at play? One way to tease apart these subtleties would be to have academics evaluate a number of detailed vignettes. It might also be interesting to determine the role of payment in ethical considerations, as ChatGPT, RAs, and proofreaders sometimes require payment and sometimes do not.

In terms of our detection design, we just considered published papers, at a top journal, and for the field of management. To understand whether these results hold more generally, it would be necessary to look at papers published in other fields, perhaps using a service like Scopus, and to look at working papers, perhaps sourced from SSRN or arXiv. In addition, it might be valuable to consider full papers instead of just abstracts, or as a middle case between abstracts and full papers, introductions might also be useful to examine.

Additionally, our results are limited to one AI detection service, so we do not know if they extend to other services, such as GPTZero. Also, it might be of interest to see if other AI-based revision services, such as Grammarly, are flagged by AI detectors too. Along these lines, it might be insightful to consider other forms of writing assistance that might be taken besides fixing grammar and rewriting text, or specific forms of rewriting text. One important dimension could be whether use of chat-based and completion-based AI tools lead to different detection rates.

Finally, and related to the last point, it would be valuable to consider a range of different prompts, especially given the sensitivity that we found to relatively small changes in prompt language. One systematic way to choose the prompts could be to hold focus groups of researchers or ChatGPT users or by having a way for researchers to vote on the prompts that are tested. Another dimension of investigation could be to see if our results are sensitive to other important features of the GPT API inputs, such as increasing the model to GPT-4, increasing the temperature to increase the hallucination rate, or by using other system prompts. Given the black box nature of LLMs, a robust empirical analysis is needed to inform policymakers at associations, journals, and conferences of the link between how AI is used and how it is flagged by detection software.

## Supporting information

S1 File Survey screenshots. (PDF)

S2 File Additional tables. (PDF)

## References

[1] Mullainathan S, Spiess J. Machine learning: an applied econometric approach. Journal of Economic Perspectives. 2017;31(2):87–106. doi: 10.1257/jep.31.2.87

[2] Athey S, Imbens GW. Machine learning methods that economists should know about. Annual Review of Economics. 2019;11:685–725. doi: 10.1146/annurev-economics-080217-053433

[3] Fudenberg D, Liang A. Predicting and understanding initial play. American Economic Review. 2019;109(12):4112–4141. doi: 10.1257/aer.20180654

[4] Farrell MH, Liang T, Misra S. Deep learning for individual heterogeneity: An automatic inference framework. arXiv preprint arXiv:201014694. 2020;.

[5] Rambachan A, et al. Identifying prediction mistakes in observational data. Harvard University. 2021;.

[6] Björkegren D, Blumenstock JE, Knight S. (Machine) Learning What Policies Value; 2022.

[7] Capra CM, Gomies M, Zhang S. The Sound of Cooperation and Deception in High Stakes Interactions. 2023;.

[8] Franchi M, Zamfirescu-Pereira J, Ju W, Pierson E. Detecting disparities in police deployments using dashcam data. In: Proceedings of the 2023 ACM Conference on Fairness, Accountability, and Transparency; 2023. p. 534–544.

[9] Salah M, Al Halbusi H, Abdelfattah F. May the force of text data analysis be with you: Unleashing the power of generative AI for social psychology research. Computers in Human Behavior: Artificial Humans. 2023; p. 100006. doi: 10.1016/j.chbah.2023.100006

[10] Beck JT, Rammage M, Jackson GP, Preininger AM, Dankwa-Mullan I, Roebuck MC, et al. Artificial intelligence tool for optimizing eligibility screening for clinical trials in a large community cancer center. JCO clinical cancer informatics. 2020;4:50–59. doi: 10.1200/CCI.19.00079 31977254

[11] Charness G, Jabarian B, List JA. Generation next: Experimentation with ai. National Bureau of Economic Research; 2023.

[12] Horton JJ. Large language models as simulated economic agents: What can we learn from homo silicus? National Bureau of Economic Research; 2023.

[13] Dietvorst BJ, Simmons JP, Massey C. Algorithm aversion: people erroneously avoid algorithms after seeing them err. Journal of Experimental Psychology: General. 2015;144(1):114. doi: 10.1037/xge0000033 25401381

[14] Deza A, Surana A, Eckstein MP. Assessment of faster r-cnn in man-machine collaborative search. In: Proceedings of the IEEE/CVF Conference on Computer Vision and Pattern Recognition; 2019. p. 3185–3194.

[15] Gajos KZ, Mamykina L. Do people engage cognitively with AI? Impact of AI assistance on incidental learning. In: 27th international conference on intelligent user interfaces; 2022. p. 794–806.

[16] Steyvers M, Tejeda H, Kerrigan G, Smyth P. Bayesian modeling of human–AI complementarity. Proceedings of the National Academy of Sciences. 2022;119(11):e2111547119. doi: 10.1073/pnas.2111547119 35275788 PMC8931210

[17] Sundar SS, Lee EJ. Rethinking communication in the era of artificial intelligence. Human Communication Research. 2022;48(3):379–385. doi: 10.1093/hcr/hqac014

[18] Tejeda H, Kumar A, Smyth P, Steyvers M. AI-assisted decision-making: A cognitive modeling approach to infer latent reliance strategies. Computational Brain & Behavior. 2022;5(4):491–508. doi: 10.1007/s42113-022-00157-y

[19] Wang X, Liang C, Yin M. The Effects of AI Biases and Explanations on Human Decision Fairness: A Case Study of Bidding in Rental Housing Markets. In: Proceedings of the Thirty-Second International Joint Conference on Artificial Intelligence, IJCAI-23, Edith Elkind (Ed.). International Joint Conferences on Artificial Intelligence Organization; 2023. p. 3076–3084.

[20] Yang N, Palma M, Drichoutis A. How Does Humanizing Virtual Assistants Affect the Propensity to Follow Their Advice? 2023;.

[21] Almog D, Gauriot R, Page L, Martin D. AI Oversight and Human Mistakes: Evidence from Centre Court. arXiv preprint arXiv:240116754. 2024;.

[22] Agrawal A, Gans J, Goldfarb A. The economics of artificial intelligence: an agenda. University of Chicago Press; 2019.

[23] Lambrecht A, Tucker C. Algorithmic bias? An empirical study of apparent gender-based discrimination in the display of STEM career ads. Management science. 2019;65(7):2966–2981. doi: 10.1287/mnsc.2018.3093

[24] Obermeyer Z, Powers B, Vogeli C, Mullainathan S. Dissecting racial bias in an algorithm used to manage the health of populations. Science. 2019;366(6464):447–453. doi: 10.1126/science.aax2342 31649194

[25] Chien CF, Dauzère-Pérès S, Huh WT, Jang YJ, Morrison JR. Artificial intelligence in manufacturing and logistics systems: algorithms, applications, and case studies; 2020.

[26] Rolf E, Simchowitz M, Dean S, Liu LT, Björkegren D, Hardt M, et al. Balancing Competing Objectives with Noisy Data: Score-Based Classifiers for Welfare-Aware Machine Learning; 2020.

[27] Zuiderwijk A, Chen YC, Salem F. Implications of the use of artificial intelligence in public governance: A systematic literature review and a research agenda. Government Information Quarterly. 2021;38(3):101577. doi: 10.1016/j.giq.2021.101577

[28] Pallathadka H, Ramirez-Asis EH, Loli-Poma TP, Kaliyaperumal K, Ventayen RJM, Naved M. Applications of artificial intelligence in business management, e-commerce and finance. Materials Today: Proceedings. 2023;80:2610–2613.

[29] Ray PP. ChatGPT: A comprehensive review on background, applications, key challenges, bias, ethics, limitations and future scope. Internet of Things and Cyber-Physical Systems. 2023;. doi: 10.1016/j.iotcps.2023.04.003

[30] Singh H, Singh A. ChatGPT: Systematic Review, Applications, and Agenda for Multidisciplinary Research. Journal of Chinese Economic and Business Studies. 2023;21(2):193–212. doi: 10.1080/14765284.2023.2210482

[31] Cowen T, Tabarrok AT. How to learn and teach economics with large language models, including GPT. Including GPT (March 17, 2023). 2023;.

[32] Daun M, Brings J. How ChatGPT will change software engineering education. In: Proceedings of the 2023 Conference on Innovation and Technology in Computer Science Education V. 1; 2023. p. 110–116.

[33] Fyfe P. How to cheat on your final paper: Assigning AI for student writing. AI & SOCIETY. 2023;38(4):1395–1405. doi: 10.1007/s00146-022-01397-z

[34] Ibrahim H, Asim R, Zaffar F, Rahwan T, Zaki Y. Rethinking Homework in the Age of Artificial Intelligence. IEEE Intelligent Systems. 2023;38(2):24–27. doi: 10.1109/MIS.2023.3255599

[35] Jungherr A. Using ChatGPT and Other Large Language Model (LLM) Applications for Academic Paper Assignments. 2023;.

[36] Malik AR, Pratiwi Y, Andajani K, Numertayasa IW, Suharti S, Darwis A, et al. Exploring Artificial Intelligence in Academic Essay: Higher Education Student’s Perspective. International Journal of Educational Research Open. 2023;5:100296. doi: 10.1016/j.ijedro.2023.100296

[37] Schmohl T, Watanabe A, Fröhlich N, Herzberg D, et al. How Artificial Intelligence can improve the Academic Writing of Students. In: Conference Proceedings. The Future of Education 2020; 2020.

[38] Shahriar S, Hayawi K. Let’s have a chat! A Conversation with ChatGPT: Technology, Applications, and Limitations. arXiv preprint arXiv:230213817. 2023;.

[39] Korinek A. Language models and cognitive automation for economic research. 2023;.

[40] Altmäe S, Sola-Leyva A, Salumets A. Artificial intelligence in scientific writing: a friend or a foe? Reproductive BioMedicine Online. 2023;. 37142479 10.1016/j.rbmo.2023.04.009

[41] Thorp HH. ChatGPT is fun, but not an author; 2023. 36701446 10.1126/science.adg7879

[42] Hill-Yardin EL, Hutchinson MR, Laycock R, Spencer SJ. A Chat (GPT) about the future of scientific publishing. Brain Behav Immun. 2023;110:152–154. doi: 10.1016/j.bbi.2023.02.022 36868432

[43] Bommasani R, Hudson DA, Adeli E, Altman R, Arora S, von Arx S, et al. On the opportunities and risks of foundation models. arXiv preprint arXiv:210807258. 2021;.

[44] Dranove D, Jin GZ. Quality disclosure and certification: Theory and practice. Journal of Economic Literature. 2010;48(4):935–63. doi: 10.1257/jel.48.4.935

[45] Jin GZ, Luca M, Martin D. Is no news (perceived as) bad news? An experimental investigation of information disclosure. National Bureau of Economic Research; 2015.

[46] Akram A. An Empirical Study of AI Generated Text Detection Tools. arXiv preprint arXiv:231001423. 2023;.

[47] Fitria TN. Grammarly as AI-powered English writing assistant: Students’ alternative for writing English. Metathesis: Journal of English Language, Literature, and Teaching. 2021;5(1):65–78.

[48] Bringula R. What do academics have to say about ChatGPT? A text mining analytics on the discussions regarding ChatGPT on research writing. AI and Ethics. 2023; p. 1–13.

