# Supplementary material for: Perceptions and detection of AI use in manuscript preparation for academic journals
Source: PLoS One. 2024 Jul 12;19(7):e0304807. doi: 10.1371/journal.pone.0304807 (PMC11244834; doi:10.1371/journal.pone.0304807)
Supplement: S1 File — (PDF) [file pone.0304807.s001.pdf]

Perceptions and detection of AI use in manuscript preparation for academic journals

Nir Chemaya[1], Daniel Martin[1,2] *

1 Department of Economics, University of California, Santa Barbara, California, USA
2 Kellogg School of Management, Northwestern University, Evanston, Illinois, USA

* daniel@martinonline.org

S1 File. Survey Screenshots.

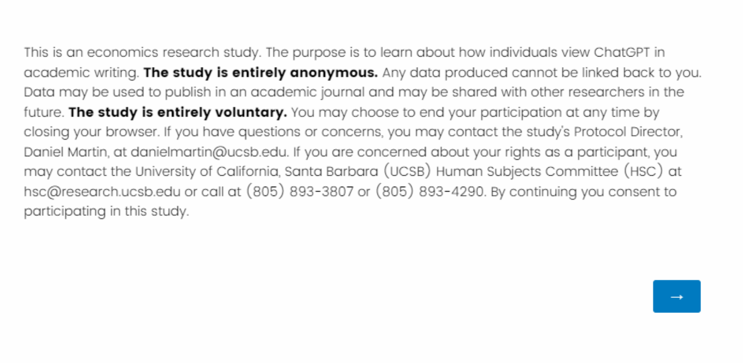

This is an economics research study. The purpose is to learn about how individuals view ChatGPT in academic writing. **The study is entirely anonymous.** Any data produced cannot be linked back to you. Data may be used to publish in an academic journal and may be shared with other researchers in the future. **The study is entirely voluntary.** You may choose to end your participation at any time by closing your browser. If you have questions or concerns, you may contact the study's Protocol Director, Daniel Martin, at danielmartin@ucsb.edu. If you are concerned about your rights as a participant, you may contact the University of California, Santa Barbara (UCSB) Human Subjects Committee (HSC) at hsc@research.ucsb.edu or call at (805) 893-3807 or (805) 893-4290. By continuing you consent to participating in this study.

→

Fig 1. Consent form.

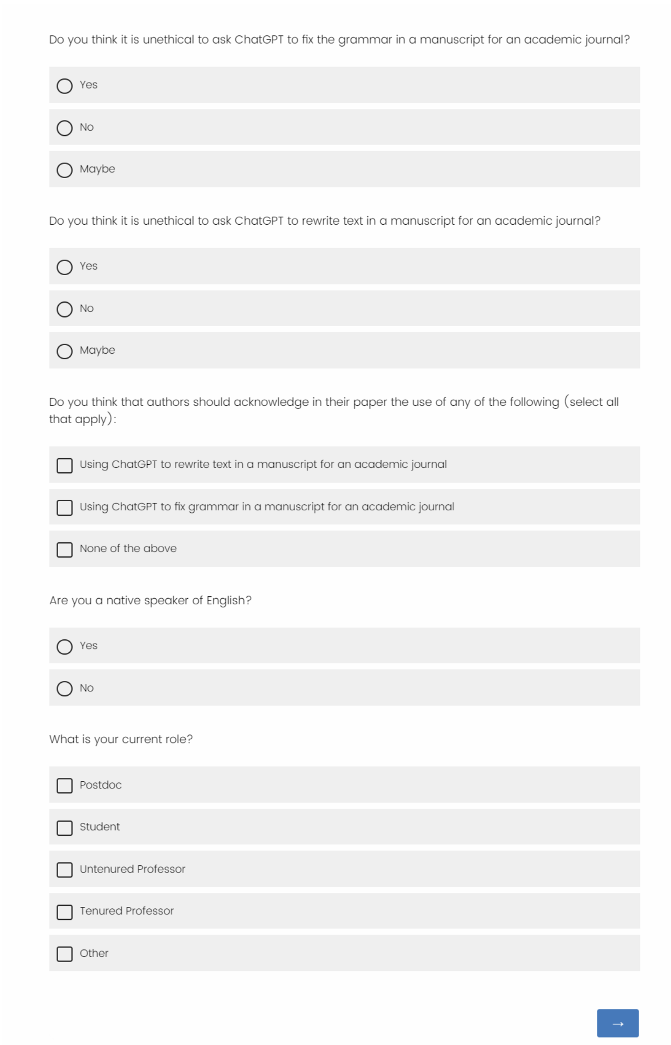

Do you think it is unethical to ask ChatGPT to fix the grammar in a manuscript for an academic journal?

- ◯ Yes
- ◯ No
- ◯ Maybe

Do you think it is unethical to ask ChatGPT to rewrite text in a manuscript for an academic journal?

- ◯ Yes
- ◯ No
- ◯ Maybe

Do you think that authors should acknowledge in their paper the use of any of the following (select all that apply):

- ☐ Using ChatGPT to rewrite text in a manuscript for an academic journal
- ☐ Using ChatGPT to fix grammar in a manuscript for an academic journal
- ☐ None of the above

Are you a native speaker of English?

- ◯ Yes
- ◯ No

What is your current role?

- ☐ Postdoc
- ☐ Student
- ☐ Untenured Professor
- ☐ Tenured Professor
- ☐ Other

→

Fig 2. Survey questions (page 1).

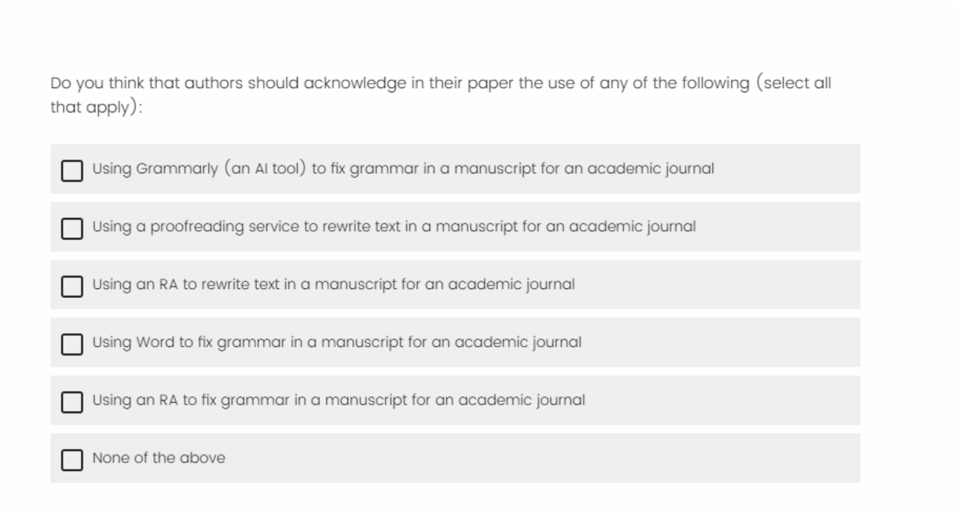

Do you think that authors should acknowledge in their paper the use of any of the following (select all that apply):

- [ ] Using Grammarly (an AI tool) to fix grammar in a manuscript for an academic journal
- [ ] Using a proofreading service to rewrite text in a manuscript for an academic journal
- [ ] Using an RA to rewrite text in a manuscript for an academic journal
- [ ] Using Word to fix grammar in a manuscript for an academic journal
- [ ] Using an RA to fix grammar in a manuscript for an academic journal
- [ ] None of the above

Fig 3. Survey questions (part 2).
