# Supplementary material for: Perceptions and detection of AI use in manuscript preparation for academic journals
Source: PLoS One. 2024 Jul 12;19(7):e0304807. doi: 10.1371/journal.pone.0304807 (PMC11244834; doi:10.1371/journal.pone.0304807)
Supplement: S2 File — (PDF) [file pone.0304807.s002.pdf]

Perceptions and detection of AI use in manuscript preparation for academic journals

Nir Chemaya[1], Daniel Martin[1,2] *

1 Department of Economics, University of California, Santa Barbara, California, USA
2 Kellogg School of Management, Northwestern University, Evanston, Illinois, USA

* daniel@martinonline.org

S2 File. Additional Tables.

Table 1 provides summary statistics for whether authors should acknowledge different forms of assistance. The top panel provides the average responses broken out by being a native English speaker or not, and the bottom panel by being a professor or not.

Table 1. The survey averages responses to whether authors should acknowledge using ChatGPT, RA, Grammarly, and Word for grammar correction, as well as ChatGPT, RA, and proofreading for text rewriting based on the respondent's role and whether the respondent is a native English speaker.

	Grammar				Rewrite			Respondents
	ChatGPT (mean)	RA (mean)	Word (mean)	Grammarly (mean)	ChatGPT (mean)	RA (mean)	Proofreading (mean)	(N)
**Native**								
Yes	0.27	0.27	0.05	0.17	0.61	0.53	0.28	83
No	0.2	0.23	0.05	0.12	0.48	0.48	0.27	188
**Total**	**0.22**	**0.24**	**0.05**	**0.14**	**0.52**	**0.5**	**0.27**	**271**
**Role**								
Professor	0.2	0.21	0.04	0.1	0.48	0.47	0.2	172
Student or Postdoc	0.25	0.3	0.07	0.2	0.59	0.56	0.38	99
**Total**	**0.22**	**0.24**	**0.05**	**0.14**	**0.52**	**0.5**	**0.27**	**271**

Table 2 shows the results of asking if it is unethical to use ChatGPT to correct the grammar or rewrite the text in a manuscript for an academic journal. For fixing grammar, there is a consensus that it is ethical to use ChatGPT, with 94% of the researchers replying that it is not unethical. However, for rewriting, this becomes more open questions with only 61% respondents thinking that is not unethical. We allow respondents to answer "maybe" to these questions to identify which researchers are uncertain about this question. However, in our analysis, we categorize respondents into those who believe it is ethical and those who either answered that it is unethical or indicate that it is "maybe" unethical.

Table 2. The survey averages responses to the questions: "Do you think it is unethical to ask ChatGPT to fix grammar (rewrite text) in a manuscript for an academic journal?" based on one's role and whether they are a native English speaker.

	Unethical Grammar (mean)	Unethical Rewrite (mean)	Respondents (N)
**Native**			
Yes	0.07	0.46	83
No	0.05	0.36	188
**Total**	**0.06**	**0.39**	**271**
**Role**			
Professor	0.06	0.38	172
Student or Postdoc	0.05	0.39	99
**Total**	**0.06**	**0.39**	**271**

When we compare native English speakers to non-native ones, we find that there is not a significant difference in perceptions of ethics around fixing grammar: 7% compared to 5% (two-sample test of proportions yields p=0.211). However, when it comes to rewriting text, 46% of native English speakers think that it is unethical to use ChatGPT, compared to 36% of non-native speakers. A two-sample test of proportions yields $p = 0.121$ on a two-tailed test or $p = 0.060$ on the one-tailed. When we compare professors to students and postdocs, we do not find any significant difference for either fixing grammar and rewriting text (for grammar p=0.76 and for rewrite p=0.977).

Finally, Table 3 shows the perceptions of respondents regarding whether one should report the use of the ChatGPT for assistance in manuscript preparation. This includes those who consider it ethical to use it and those who believe it to be unethical or are unsure (answered "maybe"). A significant difference exists among these groups. When researchers perceive it as ethical to use ChatGPT, only 21% (30%) believe that reporting is necessary when using it for grammar correction (rewriting). In contrast, researchers who view it as unethical or are uncertain about its ethics have a higher percentage, with 47% (87%) believing that reporting is necessary. This difference among the groups is significant. A two-sample test of proportions yields p=0.017 (0.0001).

Table 3. The average perception of researchers regarding whether authors should report using the ChatGPT for assistance in manuscript preparation is grouped by whether the respondent thinks it is unethical to use it to fix grammar or rewrite the academic text.

	Acknowledge (mean)	Respondents N
**Grammar**		
Unethical (Yes or Maybe)	0.47	15
Unethical (No)	0.21	256
**Total**	**0.22**	**271**
**Rewrite**		
Unethical (Yes or Maybe)	0.87	105
Unethical (No)	0.3	166
**Total**	**0.52**	**271**

Table 4. AI disclosure policy for several major publishers as of November 18, 2023.

Publisher	Disclosure	Link
Science	Mandatory	https://www.science.org/content/page/science-journals-editorial-policies
Elsevier	Mandatory	https://www.elsevier.com/about/policies-and-standards/publishing-ethics
Springer Nature	Voluntary	https://www.nature.com/nature/editorial-policies
Taylor & Francis	Mandatory	https://newsroom.taylorandfrancisgroup.com/
JAMA Network	Voluntary	https://jamanetwork.com/journals/jama/fullarticle/2801170
American Chemistry Society (ChemArxiv)	Mandatory	https://axial.acs.org/publishing/new-chemrxiv-policy-on-the-use-of-ai-tools
International Committee of Medical Journal	Mandatory	https://www.icmje.org/icmje-recommendations.pdf
World Association of Medical Editors	Mandatory	https://wame.org/page3.php?id=106
International Conference on Machine Learning	Mandatory	https://www.acm.org/publications/policies/new-acm-policy-on-authorship
